# Acceptance of Mixed Gambles Is Sensitive to the Range of Gains and Losses Experienced, and Estimates of Lambda (λ) Are Not a Reliable Measure of Loss Aversion: Reply to André and de Langhe (2020)

**DOI:** 10.1037/xge0001054

**Published:** 2021-12

**Authors:** Lukasz Walasek, Timothy L. Mullett, Neil Stewart

**Affiliations:** 1 Department of Psychology, University of Warwick; 2 Warwick Business School, University of Warwick

**Keywords:** loss aversion, decision by sampling, prospect theory, modeling, accept–reject task

## Abstract

Walasek and Stewart (2015) demonstrated that loss aversion estimated from fitting accept–reject choice data from a set of 50–50 gambles can be made to disappear or even reverse by manipulating the range of gains and losses experienced in different conditions. André and de Langhe (2020) critique this conclusion because in estimating loss aversion on different choice sets, Walasek and Stewart (2015) have violated measurement invariance. They show, and we agree, that when loss aversion is estimated on the choices common to all conditions, there is no difference in prospect theory’s λ parameter. But there are two problems here. First, while there are no differences in λs across conditions, there are very large differences in the proportion of the common gambles that are accepted, which André and de Langhe chose not to report. These choice proportion differences are consistent with decision by sampling (but are inconsistent with prospect theory or any of the alternative mechanisms proposed by André & de Langhe, 2020). Second, we demonstrate a much more general problem related to the issue of measurement invariance: that λ estimated from the accept–reject choices is extremely unreliable and does not generalize even across random splits within large, balanced choice sets. It is therefore not possible to determine whether differences in choice proportions are due to loss aversion or to a bias in accepting or rejecting mixed gambles. We conclude that context has large effects on the acceptance of mixed gambles and that it is futile to estimate λ from accept–reject choices.

In the accept–reject task, people are presented 50–50 gambles offering a monetary gain and a loss and asked whether they accept or reject the opportunity to play. For example, consider a 50–50 chance to win $20 or lose $10. If people reject this gamble, we say that they are loss averse, because the $10 loss is looming larger than the $20 gain. Walasek and Stewart (2015) manipulated the ranges of gains and losses in the choice set and showed that loss aversion, as measured by prospect theory’s λ parameter, disappears or reverses in a way predicted in advance by decision by sampling theory (DbS; Stewart et al., 2006). André and de Langhe (2020) present a critique of this result. The focal claim made by André and de Langhe is that Walasek and Stewart (2015) violated measurement invariance by estimating loss aversion on different gambles in different conditions.

We agree that the approach taken in Walasek and Stewart (2015) of comparing estimates of λ across conditions was problematic because λ was estimated on different choices in different conditions. However, in this two-part reply, we show that a model-free analysis using simple accept proportions on a common set of gambles shows strong context sensitivity, one that is entirely consistent with the predictions of DbS. We also draw on our own recent work to show that the accept–reject method is not suitable for determining whether shifts in acceptance rates are due to loss aversion (λ) or a bias to accept or reject mixed gambles, irrespective of the gains and losses on offer. In the second part of this reply, we expand on this issue and show that results of André and de Langhe’s simulations are a special case of a much more general, and worrying, issue. More specifically, we argue that the issue is not about the experimental design in Walasek and Stewart (2015), as André and de Langhe claim, but with the parameter estimation procedure. This issue affects all research that relies on estimating parameters for, for example, risk aversion, loss aversion, and probability weighting from choice behavior.

## A Model-Free Analysis of Choice Proportions

André and de Langhe reanalyzed data from Walasek and Stewart (2015), focusing on gambles that were shared between the conditions (i.e., common gambles), thus avoiding violations of measurement invariance. They found that λ did not differ between the conditions when estimated on the common choices and therefore concluded that the Walasek and Stewart (2015) experiments “should not be taken as evidence that loss aversion can disappear and reverse.”

Here we present a model-free analysis on those same common gambles, which leads to a rather different conclusion. We measure the effect of our range manipulation by simply counting the proportion of accept choices for the gambles that are common across experimental conditions (which is equivalent to the area under the indifference curve method from Walasek & Stewart, 2019, 2021; see also Pachur & Kellen, 2013, for other operationalizations). For now, we also note that this could be taken as an alternative measure of loss aversion if not for the fact that acceptance rates may also reflect status quo bias (Gal, 2006). We return to this issue shortly.

**Figure 1 fig1:**
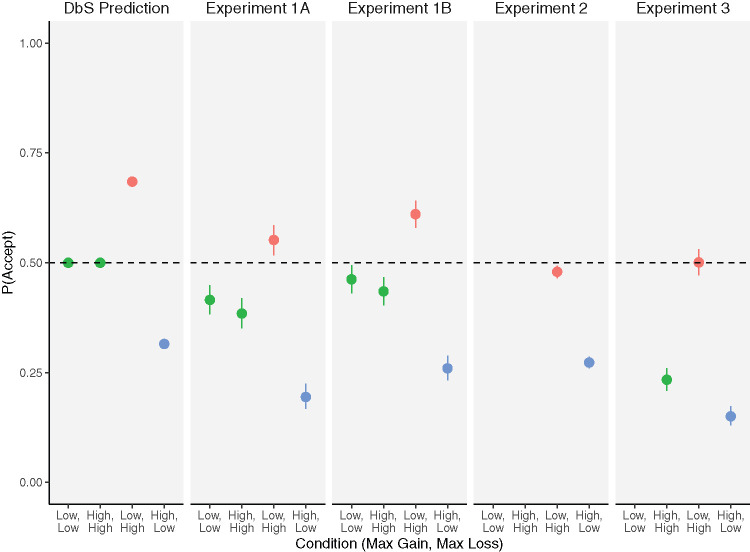
Choice Proportions Predicted by the Decision by Sampling and Proportions Found in the Four Experiments Reported by Walasek and Stewart (2015) *Note.* Leftmost panel: Decision by sampling predictions about accept proportions as a function of maximum gain and maximum loss in the choice set. Remaining panels: Accept proportions in all four experiments reported by Walasek and Stewart (2015). Code to reproduce this figure is available at https://github.com/neil-stewart/loss_aversion_common_gambles. DbS = decision by sampling theory. See the online article for the color version of this figure.

Walasek and Stewart (2015) varied the ranges from which gains and losses were drawn across conditions. In DbS, the subjective magnitude of a gain is derived from a series of comparisons with other recently experienced gains, and the subjective magnitude of a loss is derived from a series of comparisons with other recently experienced losses. The intuition as to how a range manipulation should influence acceptance rates is as follows. Consider an accept–reject decision for a 50–50 gamble offering either a loss of £10 or a gain of £10. A gain of £10 seems larger when the range of gains experienced runs from £0 to £20, where it is larger than half of the gains people experience, than £0 to £40, where it is larger than only one quarter of the gains people experience. Similarly, a loss of £10 seems larger (i.e., a bigger loss) when the range of losses experienced is £0 to £20 rather than £0 to £40. The left panel of Figure 1 shows the predictions of DbS regarding choice proportions. In the symmetrical low maximum gain, low maximum loss condition (e.g., where both losses and gains range from £0 to £20), the £10 gain and £10 loss will have similar subjective values, and people will be indifferent between accepting and rejecting this gamble. In the symmetrical high maximum gain, high maximum loss condition (e.g., where both losses and gains range from £0 to £40), people will also be indifferent by the same logic. But in the asymmetric conditions, where the maximum gain is high (£40) and the maximum loss is low (i.e., £20), the £10 loss will have a larger subjective magnitude than the £10 gain, and people will be rejecting the gamble. Finally, in the condition where the maximum gain is low (£20) and the maximum loss is high (£40) the £10 loss will have a smaller subjective magnitude than the £10 gain, and people will accept the gamble.[Fig fig1]

André and de Langhe have calculated these choice proportions during the peer-review process but chose not to describe this pattern and mention only that prospect theory’s λ, when estimated on the common choices, does not differ between conditions. However, we think that the large differences in choice proportions are important because they are consistent with DbS (which makes direct predictions about choice proportions). Furthermore, the choice proportion pattern is not consistent with the three alternative mechanisms that André and de Langhe propose (because only in DbS is subjective value a function of other gains and losses in the task).

The limitation of using choice proportions is that people’s willingness to accept mixed lotteries cannot be attributed to loss aversion alone. One could argue that the differences described above are a product of variation in a bias to accept or reject mixed lotteries—a general tendency to reject lotteries without asymmetric weighting of gains and losses. For example, one cannot tell whether the tendency to reject a 50–50 chance to win $20 or lose $10 is because the loss of $10 looms large due to loss aversion or is because of a bias to reject all mixed gambles, irrespective of the exact gain and loss on offer. Parenthetically, we do not think the effect should be dismissed as a status quo bias, where people prefer the status quo of not playing the gamble. Although it is an empirical question, we think people would still be averse to playing these mixed gambles even if they are presented as an active choice between the gamble or zero, rather than as an acceptance of the gamble. Further, we do not think the effect should be dismissed because it could be a bias rather than loss aversion. What is required is a model to explain why the acceptance rates for these gambles are so strongly affected by the range of gains and losses on offer. Only a model where the wider experience of gains and losses affects the decision to accept the gain/loss pair in a specific question, such as DbS, can do this.

In the second part of this reply, we summarize our recent work showing how bias and loss aversion cannot be reliably estimated using responses on the accept–reject task.^1^

## Estimates of **λ**

In the second part of our reply, we consider loss aversion as operationalized by prospect theory’s λ. Walasek and Stewart (2015) estimated λ could drop to 1 (meaning loss aversion had disappeared) or below 1 (a reversal of loss aversion). However, André and de Langhe (2020) show that when λ is estimated using only the gambles that appeared in all conditions, to avoid violating measurement invariance, there are no significant changes in λ across conditions. How can we see no difference in λ on the common gambles but see such a large difference in the probability of accepting the common gambles?

This null result can be attributed to the poor recoverability of the λ parameter. Simulation and recovery analyses have shown that this is the least reliable parameter within prospect theory (Broomell & Bhatia, 2014). In our own work (Walasek & Stewart, 2021), we performed a parameter recoverability exercise for the accept–reject task. We found that the bias parameter governing the overall tendency to reject/accept gambles and the λ parameter are highly correlated. As a result, both parameters suffer from poor recoverability, which led us to the conclusion that “you cannot accurately estimate an individual’s loss aversion using an accept–reject task” (title). Poor recoverability is likely to be exacerbated by choice sets that are less varied or contain fewer unique gambles, both of which are properties of fitting models only using the common gambles.

We also share André and de Langhe’s concerns about comparing λ estimates across different choice sets. Here we show that André and de Langhe’s measurement invariance critique of λ estimates is a special case of a much more serious model recovery problem. Stewart et al. (2020; see also Walasek & Stewart, 2021) show how fitting an incorrect model (so one from which the data systematically depart) leads to an omitted variable bias and, further, that this bias is different in choice sets spanning different parts of choice space. Stewart et al. (2020) show it is futile to estimate risk aversion from choices, because the risk aversion parameter estimate does not generalize even between random splits of a large and balanced choice set. Of course, all models are “wrong” to some degree as no model captures all variance in the world or perfectly predicts 100% of choices. But here we show that the magnitude of the bias introduced is so large—the same order of magnitude as the differences between individuals—as to render prospect theory parameter estimation (including λ) very troublesome.

We take the same approach as Stewart et al. (2020), fitting a version of the cumulative prospect theory to different random subsets of the large risky choice data set reported in Glöckner and Pachur (2012). From these fits, we ranked individual participants according to the magnitude of their estimated λ based on a first, randomly selected, half of choices. We then estimated λ for the same individuals on the second half of choices to assess whether their rank would change.

**Figure 2 fig2:**
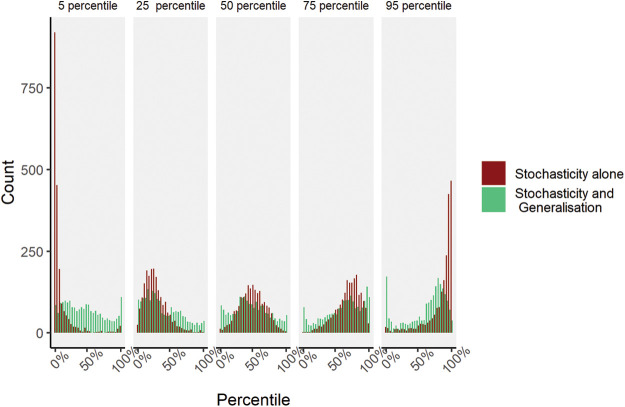
Rank Positions of Recovered λ *Note.* Each panel corresponds to the rank position of λ from the initial model fit on the subset of data (5th, 25th, 50th, 75th, and 95th). The “Stochasticity and Generalization” histograms show how people who all ranked the same in the initial set have very different ranks in the second subset. There is no relation between participants’ rank λ in the first and second sets of choices. The “Stochasticity alone” histogram shows what we would expect from noise as choices are resolved by a Bernoulli process. See the online article for the color version of this figure.

The results are shown in Figure 2. Each panel represents individuals whose λ from the first subset fit corresponds to the 5th, 25th, 50th, 75th, and 95th percentile individuals. The middle panel shows what happens when you take individuals with the median λ in the first subset of the data and plot the distribution of rank positions (in red [dark gray]) of their λ as estimated on a second subset of the data. We can see here clearly that the rank positions are extremely noisy—on some occasions, the estimate for the same person now ranks that person as one of the least loss-averse individuals; on another occasion, the same person is among the most loss-averse individuals. The same story holds true for all panels, revealing that estimates of λ are extremely unreliable. Why does this happen? Stewart et al. (2020) show that the differences between the statistical properties of the choice set, which occur even when choices are drawn from the same choice population, are such that behavioral departures from prospect theory cause considerable bias in the estimation of prospect theory parameters. Crucially, this bias varies considerably with the summary statistics of the gambles on offer and is large when compared to individual differences in parameters.[Fig fig2]

The failure of λ to generalize across even random splits of the choice set is not due simply to the stochasticity of responses. The green (light gray) distribution (see Figure 2) shows the lack of generalization that we would expect as latent choice probabilities are resolved into Bernoulli accept–reject decisions (see Stewart et al., 2020, for more details). Although generalization is not great because of stochasticity (thus we replicate earlier studies showing poor reliability of the λ parameter), it is the change of choice set that roundly kills generalization.

Thus, the argument made by Stewart et al. (2020), about the futility of estimating risk aversion, applies to loss aversion as well. The conclusion here is that it is futile to estimate λ from risky choice data. This is a profoundly worrying conclusion.

André and de Langhe attribute the problem in Walasek and Stewart (2015) to the fact that one should not generalize about parameter values estimated from different stimuli sets. Here we show that it is indeed true that parameter values do not generalize across choice sets. But if parameter estimates are completely local as André and de Langhe argue, and we cannot generalize the parameter even across random splits of a choice set, then we must ask, what is the point of the parameter?

The insight here also explains why André and de Langhe were able to make loss aversion to disappear and reverse in their simulation of three models. What all these models had in common was that they incorporated some departure from prospect theory. Thus, the data generated from these models created an omitted variable problem for the version of prospect theory being used to recover λ, which had differential effects in choice sets with different ranges of gains and losses.

## Conclusion

We agree with André and de Langhe that it is not appropriate to estimate prospect theory’s λ from different choice sets. Here we show a much more general problem: λ does not generalize across even random splits of a choice set. Thus, Walasek and Stewart’s error was in assuming that the λ parameter could be used to abstract something of an individual’s differential sensitivity to gains and losses from different choice sets—it cannot. Since the publication of the 2015 paper, Walasek and Stewart have made this point very clear in two publications (Walasek & Stewart, 2019, 2021). In this response, we extend André and de Langhe’s critique from just Walasek and Stewart (2015) to all empirical work where parameter estimates are compared across different choice sets.

We have demonstrated a large and reliable effect of range manipulation on people’s willingness to accept or reject mixed gambles. There are very large differences in acceptance rates for the gambles that are common between different conditions in the design used by Walasek and Stewart (2015). It would be misleading to omit to report this result and focus only on the null result for λ, because this result clearly implicates the role of the wider experience of gains and losses in accept–reject decisions for specific mixed gambles.

In sum, the tendency to reject 50–50 mixed gambles can be made to disappear or reverse. This was predicted in advance by decision by sampling but not prospect theory. It is now less clear how this tendency should be decomposed into loss aversion and a bias toward rejecting mixed gambles. These individual mechanisms cannot be separately or reliably estimated from people’s choices in the accept–reject task (Walasek & Stewart, 2021).

## Footnotes

1 In a personal communication, André and de Langhe propose that loss aversion and status quo bias can be separated in a model-free analysis by comparing the rate with which aggregate rejection rates increase for common gambles as the amounts of money at stake increase. They show that these rates are not different between the conditions in experiments by Walasek and Stewart (2015), which goes against the argument that range manipulations influence loss aversion. The issue of differentiating status quo (or bias) and loss aversion in the accept–reject task is the focus of the rest of our reply.
