# Correction to Walasek, Mullett, and Stewart (2021)

**DOI:** 10.1037/xge0001200

**Published:** 2022-01

In the article “Acceptance of Mixed Gambles Is Sensitive to the Range of Gains and Losses Experienced, and Estimates of Lambda (λ) Are Not a Reliable Measure of Loss Aversion: Reply to André and de Langhe (2021)” by Lukasz Walasek, Timothy L. Mullett, and Neil Stewart (*Journal of Experimental Psychology: General*, 2021, Vol. 150, No. 12, pp. 2666–2670. doi: 10.1037/xge0001054), the year of publication was changed from 2020 to 2021 in the article title, abstract, introduction, and the first paragraph of the Estimates of Lambda section to reflect the correct final publication year. The year of publication and complete citation information for Andre & de Langhe in the References list were included.

The online version of this article has been corrected.

